# An eye-tracking study on the effect of teacher image in Chinese teaching short videos

**DOI:** 10.3389/fpsyg.2026.1748769

**Published:** 2026-05-26

**Authors:** Hongming Dong, Xuan Jin, Jiaya Gong, Xuehang Dong

**Affiliations:** School of International Education, Tianjin University, Tianjin, China

**Keywords:** Chinese teaching, eye-tracking, second language acquisition, short videos, teacher image

## Abstract

In teaching short videos, the teacher’s image can quickly capture students’ attention and enhance interactivity and engagement in learning. This study, using eye-tracking experiment, explores the cognitive processing characteristics of the teacher’s image in Chinese teaching short videos and its impact on learning outcomes. The experiment recruited 30 Russian international students in China. The results show that, in the process of second language acquisition, the teacher’s image of the target language is more likely to attract learners, while the teacher’s image of a non-target language is more beneficial for students in acquiring knowledge. The learners’ attention distribution toward the teacher’s image presents a three-tier structure: “the dominant gaze, supplementary lip movements, and peripheral body.” Furthermore, learners with different Chinese proficiency levels exhibit significant differences in gaze duration. Based on the above findings, this research proposes optimization suggestions for the visual elements of international Chinese teaching short videos.

## 1 Introduction

With the rapid development of educational technologies, language learning is gradually shifting from traditional classroom settings to student-centered, multimodal learning environments (Manca, 2020; Barrot, 2022; Li and Wang, 2024) that emphasize autonomous learning (Chik and Ho, 2017; Reinders and Benson, 2017; Godwin-Jones, 2019; Huang et al., 2022; Perry et al., 2023). Among various multimedia learning environments, short videos—due to their appealing filming styles, unique visual layouts, and concise content presentation—are reshaping learners’ approaches to language learning (Pittman and Reich, 2016; Corbeil et al., 2021; Hug, 2021; Richter et al., 2022; Aslan and Sirojitdinovna, 2025).

In teaching short videos, the most visually salient element for attracting students’ attention is the teacher image, which plays a crucial role in stimulating learning interest and enhancing instructional effectiveness (Yu, 2022). The teacher image refers not only to the teacher’s appearance but also to facial expressions, ethnicity, voice and intonation, and body language (Liu, 2005; Moussu and Llurda, 2008; R’amila, 2015; Aslan and Thompson, 2017; Yu, 2022). Empirical studies show that talking-head videos, which feature the teacher’s image, significantly improve learners’ perceived closeness, motivation, and comprehension compared to audio-only or slide-based explanations (Guo et al., 2014; Yu et al., 2021). Meanwhile, when learners watch teaching short videos, they simultaneously receive information from auditory (voice) and visual (teacher’s face, gestures, subtitles) channels. This information processing is constrained by limited attentional resources and cognitive load (Sweller et al., 2022). When visual and auditory information is presented simultaneously, the visibility of the teacher image triggers a “social presence effect,” thereby enhancing learning motivation and the depth of cognitive processing (Yu et al., 2021; Li and Wang, 2024).

The teacher image is closely related to learners’ cognitive load and influences how students process and comprehend information during learning. Studies indicate that teacher presence significantly affects learners’ cognitive load. For example, compared with videos without teacher presence, videos featuring teacher presence can effectively reduce learners’ cognitive load (Chen and Wu, 2015). Videos with teacher presence can attract students’ attention, enhance their interest and satisfaction in dealing with challenging tasks, and promote learning transfer (Wang et al., 2020). Such teacher presence can elicit learners’ social responses, increase active cognitive processing, and thereby improve learning outcomes (Mayer, 2014).

However, Sweller et al. (2022), in their cognitive load theory, point out that when learners’ working memory resources are consumed by redundant visual information, learning efficiency may decrease. Therefore, some studies suggest that excessive visual presence of the teacher may impose additional cognitive load, especially when information channels are competing intensively (Skulmowski and Xu, 2022). Some studies argue that teacher presence may distract learners and increase cognitive load (Harp and Mayer, 1998; Lyons et al., 2012). Moreover, cognitive load is determined not only by whether the teacher appears but also by how the teacher is presented. Continuous appearance of the teacher in videos may increase cognitive load, whereas intermittent appearance can significantly reduce it (Yi et al., 2019). Thus, the role of the teacher image in teaching short videos still requires further exploration.

The teacher image is also closely related to learners’ perceptions of linguistic authority and cultural identity, particularly in the process of second language acquisition. Giles (1979) linguistic authority theory and Rosch (1975) prototype theory suggest that learners often regard target language teachers as the “prototypical” representatives of the target language and attribute to them higher authority, leading to attentional and affective biases. Specifically, the facial and lip features of the target language teacher may trigger an “authority prioritization effect,” directing learners’ early visual attention toward the teacher (Taylor, 2003). However, non-target language teachers, as “successful second language learners,” also possess certain advantages. The concept of attribute similarity (Schunk et al., 1987; Bandura, 1997) explains that when learners observe role models who share similar characteristics with themselves (such as gender, ethnicity, age), their self-efficacy and likelihood of task success increase. Moreover, people tend to be more attracted to those who resemble themselves (Berscheid and Walster, 1969; Byrne and Nelson, 1965; Kim and Wei, 2011). This attraction may influence real-world learning performance. Therefore, the roles of target language teachers and non-target language teachers in second language acquisition still require further investigation.

In recent years, eye-tracking technology has been widely applied to video-based learning to explore learners’ attention distribution and information processing pathways (Kredel et al., 2017; Xu et al., 2025). Eye-tracking research shows that learners’ gaze is often concentrated on the teacher’s face—especially the lips and eyes—because these areas provide important cues for pronunciation and intonation recognition in language learning (Conklin and Pellicer-Sánchez, 2016). Li (2024) found that the teacher’s facial region, particularly the eyes and lips, serves as a prominent attentional focus in language learning videos, and its dynamic changes can activate the mirror neuron system (Caramazza et al., 2014; Iacoboni and Dapretto, 2006), thereby facilitating embodied learning through “observation–imitation” mechanisms. This means that the teacher’s facial expressions, lip movements, and gaze not only transmit information but also participate in the synchronous processing of language perception and motor representation. Rich facial expressions (e.g., smiling or nodding) have been shown to prolong gaze duration and evoke stronger emotional engagement, that is highly beneficial for learning (Zhan and Zhu, 2025). In addition, Liu (2021) indicated that under the text-image combined condition, learners distribute their visual attention more evenly between text and images. Wang and An (2022) adopted eye-tracking technology and found that proficient second language Chinese readers exhibit a more efficient lexical processing pattern. Qiu et al. (2026) found that intermediate second language Chinese learners tend to read the text first and then view the images, while advanced learners demonstrate a more flexible pattern of alternating gaze between text and images. Therefore, owing to the advancement of eye-tracking technology, a deeper understanding can be gained regarding how learners’ attention is distributed across different components of the teacher’s image.

This study, therefore, adopts an eye-tracking experimental approach to systematically analyze the cognitive processing characteristics associated with the teacher image in Chinese teaching short videos and its impact on learning outcomes in second language acquisition. The specific objectives include: (1) revealing differences in visual attention allocation among learners toward teachers with different cultural identities (target language teacher vs. non-target language teacher); (2) exploring the role of facial and lip information in cognitive processing; and (3) analyzing differences in learning outcomes under different teacher images. By achieving these objectives, the study aims to provide empirical support for the design of Chinese teaching short videos and promote the personalized and sustainable development of language learning.

## 2 Methodology

### 2.1 Participants and procedure

This study recruited 30 Russian Chinese learners as experimental subjects. Given the noticeable differences in the typical visual representations of Russian and Chinese teachers, particularly in terms of facial features and expressions, Russian participants were selected as they are well-suited for examining how target versus non-target teacher images influence visual attention allocation. Based on the “International Chinese Language Education Proficiency Level Standards,” they were divided into two groups: the Advanced Level Group (*n =* 14, Chinese proficiency level 5–6) and the Beginner Level Group (*n =* 16, Chinese proficiency level 3–4). Before the experiment, the researchers provided all participants with a detailed explanation of the research plan (including experimental content, duration, and data usage) and the potential for mild discomfort (such as short-term visual fatigue or simple cognitive task pressure). After confirming that participants fully understood the information, they voluntarily signed an informed consent form. Participants were also clearly informed that they had the right to terminate their participation in the experiment at any time, without the need to provide a reason, and that exercising this right would not have any negative consequences.

The experimental procedure consists of two main stages: the preparation stage and the experimental stage. The preparation stage includes four steps: first, assigning a trial number to each participant and registering their basic information; second, explaining the experimental process and ensuring participants sign the informed consent form; third, administering a pre-test and allowing participants to become familiar with the experimental equipment and environment; and finally, performing eye calibration to ensure the accuracy of data collection. The experimental stage consists of three tasks: first, playing Teaching Video 1 and asking participants to complete the corresponding scale and test items; second, playing Teaching Video 2 and having participants complete the corresponding scale and test items; and finally, playing Teaching Video 3 and asking participants to complete the scale and test items again.

### 2.2 Instruments and experiment design

**Figure 1:**
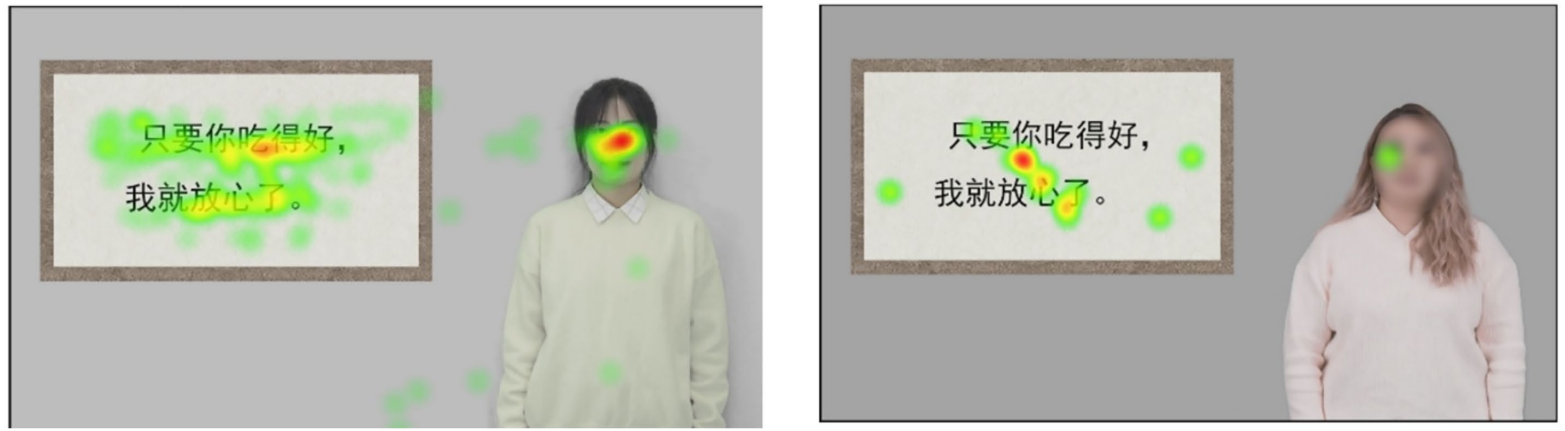
Typical eye-tracking heatmaps of the participants.

This experiment is based on the “International Chinese Language Education Proficiency Level Standards” (Ministry of Education of the People’s Republic of China and National Language Commission of China, 2021). The experiment selects two groups of knowledge points: Level 3–4 (Beginner) and Level 5–6 (Intermediate-Advanced) Chinese proficiency. The Beginner group focuses on basic grammar items such as “不敢当,” “只要…就…,” and “就算…,” selected from the core modules of HSK标准教程3-4; the Intermediate-Advanced group selects more complex sentence structures such as “X就X吧,” “X就X了,” and “只有…才…,” sourced from HSK标准教程5 and the 发展汉语·高级综合 grammar syllabus. To investigate the teacher’s image, this experiment creates two versions of teaching videos for each knowledge point: one with a target language teacher (a female teacher from China Figure 1 left) and one with a non-target language teacher (a female teacher from Russia Figure 1 right). To ensure consistency in the sound variable, all versions of the videos featuring different teacher images use the same audio recording; the teacher’s lip movements were also adjusted through post-production to align with the audio and maintain audiovisual synchrony. To eliminate interference from irrelevant variables, the video duration, script, teacher’s voice, and speaking speed remain consistent, while the background, subtitles, and position are standardized across modalities. Each knowledge point is followed by two pre-test and post-test questions.

The experiment uses the Tobii Pro S1200 desktop eye tracker, with a sampling rate of 1,200 Hz, a 17-inch screen size, and a resolution of 1280*1024 pixels. It automatically records participants’ eye movement data as they view the stimulus material. The experiment divides the overall video screen into three areas of interest: the PPT area, the teacher’s image area, and the subtitle area. To further explore the learners’ attention patterns toward the teacher’s image, the teacher’s image is further subdivided into three sub-regions: gaze area, lip movement area, and body area.

### 2.3 Data analysis and findings

#### 2.3.1 Overall attention allocation

To explore the impact of teacher image type on overall attention allocation in the visual scene, this study conducted an independent sample t-test on the eye-tracking data of six indicators in the total visual scene of international Chinese teaching short videos for the target language teacher image group and the non-target language teacher image group. The results show that the median total fixation time of learners watching the target language teacher image video was overall greater than that of learners watching the non-target language teacher image video (62,475 ms > 50,412 ms). The pupil diameter of learners watching the target language teacher image video was overall larger than that of learners watching the non-target language teacher image video (3.1 mm > 2.8 mm). The eyelid movement amplitude of learners watching the target language teacher image video was overall larger than that of learners watching the non-target language teacher image video (9.5 mm > 8.4 mm) (see Table 1).

**Table 1:** Comparison of overall attention allocation.

Eye-tracking indicators	Target teacher Mdn (IQR)	Non-target teacher Mdn (IQR)	U	Z	*p*
First fixation duration (ms)	624 (383–772)	512 (336–703)	86.5	−1.1	0.289
Total fixation duration (ms)	62,475 (53445–68,223)	50,412 (46014–62,418)	51	−2.5	0.011
Average fixation duration (ms)	845 (811–1,012)	850 (506–1,102)	99	−0.5	0.589
Fixation count (count)	208 (183–241)	190 (126–227)	82	−1.2	0.212
Pupil diameter (mm)	3.1 (2.8–3.3)	2.8 (2.5–3.0)	45	−2.8	0.005
Eyelid movement amplitude (mm)	9.5 (8.8–10.3)	8.4 (7.7–8.8)	42	−2.9	0.004

Total fixation time refers to the total duration of a learner’s gaze on a specific stimulus, representing the intensity of their visual attention to that stimulus. It reflects cognitive processing depth and psychological involvement, as well as information processing difficulty, interest, or understanding needs. Learners’ total fixation time on the target language teacher image video was significantly longer than on the non-target language teacher image video. This phenomenon suggests that learners have a higher visual attention intensity and cognitive processing investment toward the target language teacher image, which may be due to the target language teacher’s language authority, cultural proximity, or teaching affinity. These factors are more likely to trigger learners’ information processing demands, interest, and expectations for understanding, leading to a prioritization of attention and deeper processing of the target language teaching context.

Pupil diameter reflects the intensity of cognitive load, while eyelid movement amplitude is an important physiological indicator of attention resource allocation and cognitive load. The pupil diameter (Z = −2.7, *p =* 0.008) and eyelid movement amplitude (Z = −3.2, *p =* 0.001) of the target language teacher image group were significantly higher than those of the non-target language teacher group. This means that teaching short videos with the target language teacher image induce stronger sustained attention and physiological arousal. However, no significant differences were found in first fixation duration (Z = −1.1, *p =* 0.289), average fixation duration (Z = −0.5, *p =* 0.589), and fixation count (Z = −1.2, *p =* 0.212), indicating that the two types of teacher images did not significantly differ in attracting initial attention or the degree of fixation dispersion in the total visual scene of the teaching video (see Table 1).

The typical eye-tracking heatmap shows that there are significant differences in the distribution of high-heat areas between the two groups of videos in the overall visual scene. The heatmap employs a color gradient to represent fixation intensity: red areas indicate the highest fixation density; yellow areas represent relatively high levels of visual attention; green areas indicate lower fixation density and areas without noticeable coloring correspond to regions that received little or no visual attention. The target language teacher image group video has more high-heat areas, covering a larger area, and these areas are distributed in clusters. In contrast, the non-target language teacher image group video has fewer high-heat areas, covering smaller areas, and these areas are scattered (see Figure 1). This indicates that different types of teacher images can affect learners’ cognitive resources, and, compared to the non-target language teacher videos, learners engage in longer periods of sustained deep processing with the target language teacher videos, which is consistent with the eye-tracking data results.

In summary, learners watching teaching videos with a target language teacher image have longer total fixation times, larger pupil diameters, and greater eyelid movement amplitudes, which significantly differ from those of the non-target language teacher image videos. This indicates that different types of teacher images can influence learners’ attention and cognition. Moreover, the target language teacher image leads to longer periods of sustained deep processing, higher cognitive load, and stronger physiological arousal for learners.

#### 2.3.2 Attention allocation across different AOIs

This study divides the overall visual scene into three main areas of interest: PPT, teacher image, and subtitles. The Mann–Whitney U test was used to perform cross-group comparisons of the two groups across these three areas of interest (PPT, teacher image, and subtitles), with the significance level set at *α* = 0.05 (two-tailed). The aim is to further investigate the impact of different teacher images (target language teacher image and non-target language teacher image) on students’ cognitive processing during the learning process in the teaching video scene.

1 PPT AOI

In the teaching video, the PPT serves as an auxiliary tool, presenting the teaching content in a visualized manner. The analysis of six eye-tracking indicators for the PPT area shows that the first fixation time, total fixation time, and average fixation time are not significant (*p* > 0.05), while pupil diameter and eyelid movement amplitude are significant (*p <* 0.01) (see Table 2). The median pupil diameter of learners watching the target language teacher image video was 3.1 mm, which was overall larger than the median pupil diameter of learners watching the non-target language teacher image video (2.7 mm). The median eyelid movement amplitude of learners watching the target language teacher image video was 9.5 mm, which was overall larger than the median eyelid movement amplitude of learners watching the non-target language teacher image video (8.4 mm). There were significant differences in pupil diameter (Z = −2.7, *p =* 0.008) and eyelid movement amplitude (Z = −3.2, *p =* 0.001) between the target language teacher image group and the non-target language teacher image group. This indicates that learners’ processing of PPT content was deeper when watching the target language teacher image video, meaning that when the target language teacher explained, learners experienced higher cognitive load or showed stronger interest in the content. This increase in interest or cognitive load prompted learners to process the information in the PPT more deeply.

2 Teacher image AOI

**Table 2:** Comparison of eye movement behavior in the PPT AOI.

Eye-tracking indicators	Target teacher Mdn (IQR)	Non-target teacher Mdn (IQR)	U	Z	*p*
First fixation duration (ms)	164 (139–202)	154 (125–221)	102	−0.4	0.678
Total fixation duration (ms)	44,987 (34380–58,694)	39,710 (15509–45,725)	76	−1.5	0.135
Average fixation duration (ms)	263 (233–335)	248 (217–329)	99	−0.5	0.589
Fixation count (count)	166 (121–206)	145 (49–189)	95	−0.7	0.48
Pupil diameter (mm)	3.1 (2.9–3.3)	2.7 (2.5–3.0)	48	−2.7	0.008
Eyelid movement amplitude (mm)	9.5 (8.8–10.6)	8.4 (7.4–8.8)	34	−3.2	0.001

The analysis of eye-tracking indicators in the teacher image area between the target language teacher group and the non-target language teacher group shows significant differences in first fixation duration (Z = −2.2, *p =* 0.031), pupil diameter (Z = −3.4, *p =* 0.001), and eyelid aperture (Z = −2.3, *p =* 0.02) (see Table 3). First fixation duration reflects learners’ initial attentional investment and the processing difficulty associated with specific information. The results show that the median first fixation duration on the teacher image area in videos with the target language teacher is 245 ms, which is significantly longer than that for the non-target language teacher. A longer first fixation duration indicates that learners require more time for initial perception and comprehension; it may also suggest that the stimulus is more attractive and can more quickly capture learners’ attention. Within the teacher image area, the median pupil diameter of learners watching the target language teacher video is 3.3 mm, which is overall larger than that of learners watching the non-target language teacher video (median *=* 2.7 mm). The median eyelid aperture of learners watching the target language teacher video is 9.5 mm, also larger than that of those watching the non-target language teacher video (median *=* 8.6 mm). The significant differences in pupil diameter (Z = −3.4, *p =* 0.001, r = 0.62) and eyelid aperture (Z = −2.3, *p =* 0.02, r = 0.43) between the two groups indicate that learners experience higher cognitive load and stronger emotional arousal when processing the teacher image of the target language teacher.

1. 3 Subtitle AOI

**Table 3:** Comparison of eye movement behavior in the teacher image AOI.

Eye-tracking indicators	Target teacher Mdn (IQR)	Non-target teacher Mdn (IQR)	U	Z	*p*
First fixation duration (ms)	245 (193–287)	164 (115–218)	60	−2.2	0.031
Total fixation duration (ms)	9,967 (2944–16,017)	6,283 (3128–10,584)	96	−0.7	0.506
Average fixation duration (ms)	448 (297–488)	317 (214–475)	82	−1.2	0.212
Fixation count (count)	23 (9–31)	17 (11–34)	91	−0.9	0.383
Pupil diameter (mm)	3.3 (2.9–3.3)	2.7 (2.5–2.8)	30	−3.4	0.001
Eyelid movement amplitude (mm)	9.5 (9.0–10.4)	8.6 (7.4–9.4)	56	−2.3	0.02

The analysis of eye-tracking indicators in the subtitle area for the target language teacher group and the non-target language teacher group shows no significant differences in total fixation duration, mean fixation duration, fixation count, pupil diameter, or eyelid aperture (*p* > 0.05) (see Table 4). This indicates that the use of either a target language teacher image or a non-target language teacher image in the instructional videos did not produce a significant impact on learners’ attention allocation to the subtitles.

Overall, learners’ behaviors when viewing instructional videos featuring teacher images show significant differences in first fixation duration, pupil diameter, and eyelid aperture, with longer first fixation durations and larger pupil diameters and eyelid apertures. In videos featuring the target language teacher image, learners require more initial time for perception and comprehension in both the PPT and teacher image areas, accompanied by higher cognitive load and stronger emotional arousal. However, in both the PPT and teacher image areas, larger pupil diameters and greater eyelid apertures do not coincide with longer total fixation duration. This suggests that the rich attributes represented by the target language teacher image do not simply increase learners’ cognitive burden; instead, they may develop a unique adaptive learning value through symbolic information compression and cross-modal enhancement effects, helping learners reconcile the tension between high cognitive load and low processing efficiency.

**Table 4:** Comparison of eye movement behavior in the subtitle AOI.

Eye-tracking indicators	Target teacher Mdn (IQR)	Non-target teacher Mdn (IQR)	U	Z	*p*
First fixation duration (ms)	173 (146–221)	151 (126–185)	73	−1.4	0.162
Total fixation duration (ms)	5,975 (1796–14,890)	4,213 (1423–11,844)	86	−0.8	0.407
Average fixation duration (ms)	192 (173–275)	204 (154–321)	101.5	−0.2	0.879
Fixation count (count)	14 (4–36)	16 (5–37)	95.5	−0.4	0.678
Pupil diameter (mm)	2.9 (2.7–3.2)	2.7 (2.5–2.9)	70	−1.5	0.127
Eyelid movement amplitude (mm)	8.7 (8.2–10.2)	7.4 (5.3–9.6)	63	−1.8	0.067

#### 2.3.3 Attention allocation in sub-regions of the teacher image AOI

**Figure 2:**
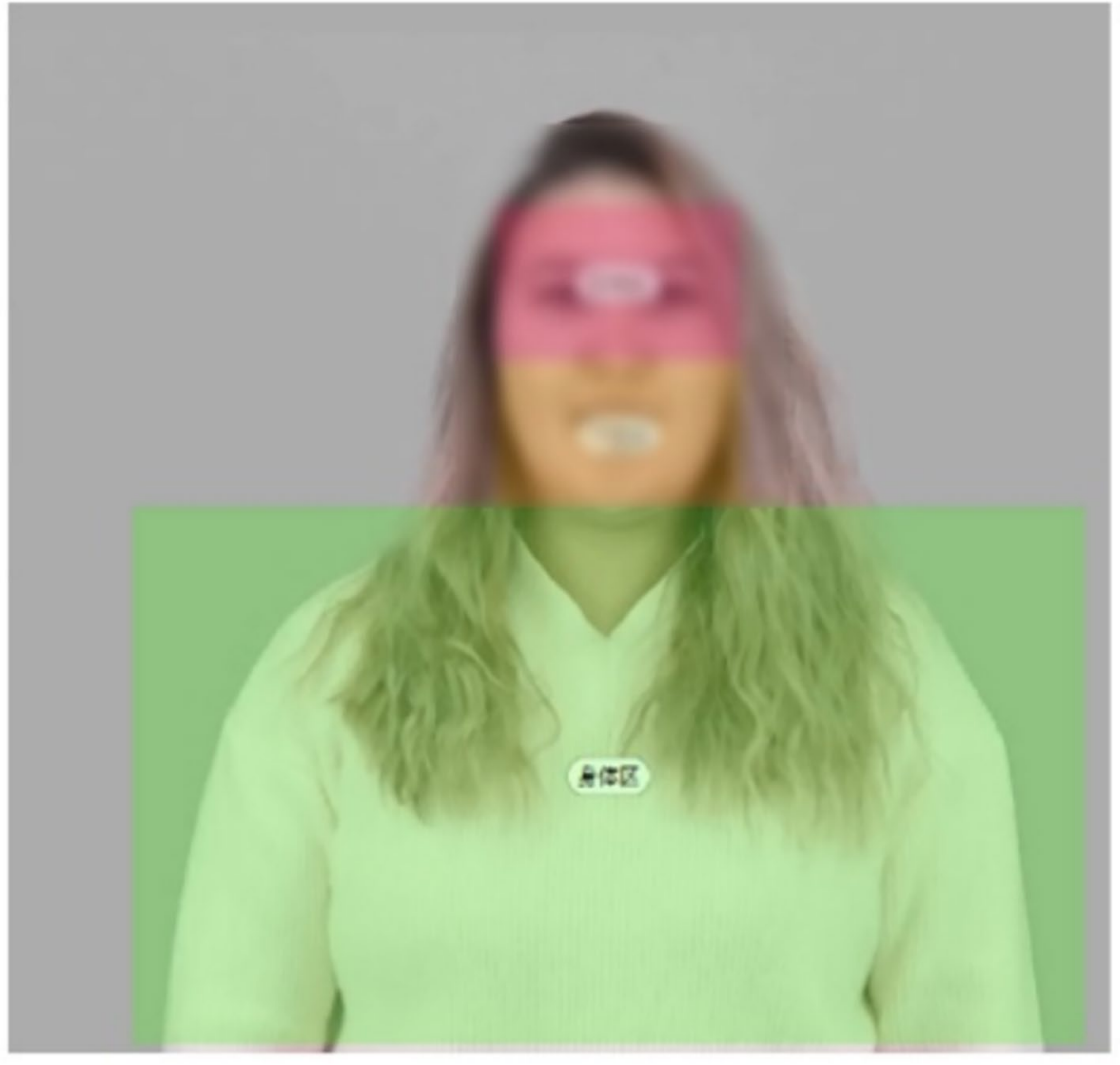
Teacher image AOI division (red: gaze area; orange: lip area; green: body area).

To further analyze learners’ attentional differences toward the two types of teacher images, this study decomposed the teacher image area into three interest areas: the gaze area (red region in Figure 2), the lip-movement area (orange region in Figure 2), and the body-language area (green region in Figure 2). To eliminate potential bias caused by differences in the size of these regions, the data were standardized prior to analysis. A Friedman test was conducted to examine differences in fixation duration across the three related interest areas (gaze/lips/body). To avoid the problem of multiple comparisons, our alpha level was adjusted using the Bonferroni correction method (three comparisons; *p =* 0.0167).

Descriptive statistics revealed that the minimum values for first fixation duration, total fixation duration, and mean fixation duration in the body-language area were all 0, indicating that some learners did not fixate on the body-language area at any point during the viewing process (see Table 5). The gaze area and lip-movement area exhibited highly concentrated visual attention: the median total fixation duration for the gaze area (48,673 ms) was approximately 2.7 times that of the lip-movement area (18,133 ms) and 215 times that of the body-language area (226 ms). Overall, learners’ visual processing of the teacher image displayed a three-tier attentional structure characterized as “gaze-dominant, lips-supporting, and body-peripheral.”

**Table 5:** Comparison of eye-tracking behavior in sub-regions of teacher image AOI.

Eye-tracking indicators	Gaze area Mdn (IQR)	Lip area Mdn (IQR)	Body area Mdn (IQR)
First fixation duration (ms)	4,592 (3095–6,288)	4,432 (2738–7,880)	159 (0–251)
Total fixation duration (ms)	48,673 (12649–120,860)	18,133 (6473–33,578)	226 (0–372)
Average fixation duration (ms)	6,087 (4423–8,825)	5,546 (3970–10,446)	167 (0–269)
Fixation count (count)	98 (42–226)	46 (16–76)	52 (18–87)
Pupil diameter (mm)	2.8 (2.7–3.2)	2.8 (2.7–3.2)	2.6 (0.0–2.9)
Eyelid movement amplitude (mm)	9.3 (8.5–9.9)	9.4 (8.3–9.9)	7.7 (0.0–9.6)

The Friedman test showed significant overall differences in fixation indicators across the gaze area, lip-movement area, and body-language area (*p <* 0.001) (see Table 6), indicating a clear hierarchical differentiation in learners’ fixation patterns on different regions of the instructional videos during language learning. Specifically, the gaze and lip-movement areas exhibited significantly higher first fixation duration, total fixation duration, and fixation count than the body-language area (*p <* 0.001). Differences between the gaze and lip-movement areas were primarily reflected in the dimension of processing depth (mean fixation duration and fixation count, *p <* 0.01). Pupil diameter and eyelid aperture showed highly consistent levels of physiological arousal in the core facial regions (gaze and lip-movement areas), with no significant differences between the two (*p* > 0.05), but both were significantly higher than those in the body-language area (*p <* 0.01).

**Table 6:** Comparison of eye-tracking behavior in sub-regions of teacher image AOI.

Eye-tracking indicators	Friedman test	*Post-hoc* comparison (Wilcoxon Z test)
First Fixation Duration (ms)	*p <* 0.001	Gaze vs. Lip: Z = −0.113, *p =* 0.910
		Gaze vs. Body: Z = −4.782, *p =* 0.000
		Lip vs. Body: Z = −4.681, *p =* 0.000
Total Fixation Duration (ms)	*p <* 0.001	Gaze vs. Lip: Z = −3.610, *p =* 0.000
		Gaze vs. Body: Z = −4.782, *p =* 0.000
		Lip vs. Body: Z = −4.681, *p =* 0.000
Average fixation duration (ms)	*p <* 0.001	Gaze vs. Lip: Z = −3.532, *p =* 0.000
		Gaze vs. Body: Z = −3.281, *p =* 0.001
		Lip vs. Body: Z = −4.627, *p =* 0.000
Fixation count (count)	*p <* 0.001	Gaze vs. Lip: Z = −3.532, *p =* 0.000
		Gaze vs. Body: Z = −3.281, *p =* 0.001
		Lip vs. Body: Z = −4.627, *p =* 0.000
Pupil diameter (mm)	*p <* 0.001	Gaze vs. Lip: Z = −1.841, *p =* 0.066
		Gaze vs. Body: Z = −2.396, *p =* 0.017
		Lip vs. Body: Z = −2.606, *p =* 0.009
Eyelid movement amplitude (mm)	*p <* 0.001	Gaze vs. Lip: Z = −0.134, *p =* 0.894
		Gaze vs. Body: Z = −2.993, *p =* 0.003
		Lip vs. Body: Z = −2.584, *p =* 0.01

Overall, learners’ attention allocation toward the teacher image exhibits a three-tier structure characterized as “the dominant gaze, supplementary lip movements, and peripheral body.” Learners devote very little attention to the body-language area and instead focus primarily on the eyes and mouth, as these regions contain essential linguistic information.

#### 2.3.4 Learning outcomes

To investigate the effects of different teacher images on learning outcomes, this study conducted paired-samples t-tests on learners’ test scores before and after watching Chinese teaching short videos in each group, as shown in Table 7.

**Table 7:** Comparison of pre-test and post-test scores.

Knowledge point	Non-target language teacher						Target language teacher					
	1	2	3	4	5	6	1	2	3	4	5	6
Z	−1.732	−1.667	−2	−2	−2.333	−1.576	−0.577	−0.577	−2.646	−1.518	−1.994	−1.667
*p*	0.083	0.096	0.046	0.046	0.020	0.115	0.564	0.564	0.008	0.129	0.046	0.096

After comparing the pre- and post-test learning outcomes of the target language teacher image group and the non-target language teacher image group, the statistical results indicate that both groups demonstrated significant improvements across multiple measurement items (*p <* 0.05). This finding suggests that instructional videos featuring either target language teachers or non-target language teachers can significantly enhance Chinese language learners’ performance, further confirming the overall effectiveness of instructional videos as a pedagogical medium for promoting Chinese language acquisition. Based on the confirmation of the general effectiveness of video-based instruction, a comparative analysis of the two types of teacher images was subsequently conducted. Specifically, the non-target language teacher group showed significant improvement in three knowledge items in the post-test, with a greater number of significant items than the target language teacher group. In sum, Chinese teaching short videos featuring non-target language teacher images demonstrate a more substantial teaching advantage compared with videos featuring target language teacher images. Overall, they appear to be more conducive to learners’ mastery of Chinese language knowledge points.

#### 2.3.5 Learners of different Chinese proficiency levels

Using the Mann–Whitney U test, eye-tracking data from the intermediate-level group and the advanced-level group were analyzed as independent samples, with Chinese proficiency used as the grouping variable (Table 8). The results show that, except for mean fixation duration—which reached a marginally significant level (*p =* 0.050)—no significant differences were found between the two proficiency groups in first fixation duration, total fixation duration, total fixation count, mean pupil diameter, or mean eyelid aperture. Specifically, learners at the beginner level tended to exhibit longer mean fixation durations, while those at the advanced level showed comparatively shorter durations.

**Table 8:** Comparison of pre-test and post-test scores.

Eye-tracking indicators	Beginner level group	Advanced level group	Z	*P*
First fixation duration (ms)	160.000 (129.3, 238.5)	158.333 (130.6, 184.4)	−0.262	0.793
Total fixation duration (ms)	5565.500 (2503.7, 13950.7)	3789.667 (1346.2, 14918.8)	−0.567	0.57
Average fixation duration (ms)	218.333 (183.0, 321.0)	162.417 (141.5, 222.5)	−1.964	0.050
Fixation count (count)	18.667 (4.7, 71.0)	11.250 (3.4, 27.3)	−1.288	0.198
Pupil diameter (mm)	2.791 (2.5, 3.1)	2.789 (2.4, 3.0)	−0.567	0.57
Eyelid movement amplitude (mm)	8.314 (7.4, 10.1)	7.845 (5.3, 10.4)	−0.742	0.458

## 3 Discussion

Based on an eye-tracking experiment, this study systematically examined the cognitive processing characteristics of teacher images in short video–based Chinese instruction and their impact on learning outcomes. The findings reveal that the teacher image is not only an important visual carrier of instructional content but also plays a central role in learners’ attention allocation, information processing, and emotional responses. Eye-tracking data demonstrate that target language teachers, owing to their cultural authority and prototypical features, occupy learners’ early attentional resources to a significantly greater extent, eliciting deeper cognitive engagement. Learners exhibit an “authority prioritization” effect during the initial fixation stage when processing the facial features of target language teachers. This aligns with linguistic authority theory (Giles, 1979) and prototype category theory (Rosch, 1975). The native speaker’s articulatory demonstration further activates the mirror neuron system (Iacoboni and Dapretto, 2006), reinforcing an embodied “observation–imitation” learning pathway. In contrast, non-target language teachers did not evoke the anticipated novelty effect; their “non-prototypical” identity may trigger cultural schema conflict, leading learners to classify them as lower-efficiency information sources during early perceptual processing, thereby reducing their perceptual salience.

In terms of information-channel allocation, learners’ attention to the subtitle area was notably reduced. Subtitle fixation time approached a near-random level, suggesting that lower-proficiency learners, restricted by their linguistic competence, were unable to process auditory and visual information simultaneously and thus automatically downregulated attention to subtitles. This finding supports the redundancy effect described in cognitive load theory (Sweller et al., 2022). Meanwhile, learners’ visual attention exhibited a clear hierarchical structure: the facial region—particularly the gaze and lip areas—served as the primary focus of cognitive resources. This “face-priority” pattern indicates that under conditions of resource constraints, learners automatically select the information most relevant to language comprehension (such as lip movements, facial expressions, and gaze direction) while reducing attention to less essential cues (such as body movements or background elements), thereby maximizing processing efficiency. This is in line with existing literature, faces and gaze direction can automatically capture attention (Frischen et al., 2007), and such processing is highly efficient and requires minimal cognitive resources. Specifically, when processing verbal information, learners dynamically allocate their attention to different facial regions: the eye area is used to acquire social cues and gaze direction, while the mouth area serves to extract visual speech information (Võ et al., 2012; Thompson et al., 2004).

Notably, although target language teachers enjoy a perceptual advantage in attracting early attention, the instructional videos featuring non-target language teachers produced more substantial learning gains. Learners in the non-target language teacher group demonstrated superior performance in the post-test. This outcome may be attributable to two factors. First, non-target language teachers typically position themselves as “successful second language learners,” possessing greater empathy and strategy awareness. Their learning experiences enable them to anticipate learners’ difficulties and explicitly convey metacognitive strategies and learning methods, thereby constructing effective cognitive scaffolding. Second, their language output tends to align more closely with learners’ interlanguage level, featuring moderate speech rate and appropriate lexical and syntactic complexity. This corresponds to Krashen’s (1982) “comprehensible input” hypothesis, which maintains linguistic accuracy while reducing cognitive load. Furthermore, the successful learning experiences and identity characteristics of non-target language teachers exert an affective motivational effect, reducing learners’ anxiety when dealing with more advanced content and enhancing self-efficacy and learning motivation. These factors collectively contribute to the sustained positive impact on learning outcomes.

## 4 Conclusion

In summary, this study, through an eye-tracking experiment, reveals the multidimensional role of teacher images in short video–based instruction. Differences between target language and non-target language teacher images significantly influence learners’ behaviors when watching instructional videos. Overall, target language teacher images more readily attract learners’ attention: learners exhibit longer perceptual–processing times, higher cognitive load, and stronger emotional arousal in the overall video frame, the PPT interest area, and the teacher image interest area when viewing videos featuring target language teachers. In addition, learners’ visual attention to different subregions of the teacher image displays a clear hierarchical structure, characterized as “gaze-dominant, lips-supporting, and body-peripheral.” The gaze and lip-movement areas, functioning as dual hubs for linguistic and social information, receive substantially greater cognitive resources, whereas fixation on the body-language area approaches near-zero levels. Learning outcome data further show that instructional videos featuring non-target language teacher images demonstrate a more substantial teaching advantage compared with those featuring target language teachers, thus being more conducive to learners’ mastery of Chinese language knowledge. Moreover, learners of different Chinese proficiency levels exhibit significant differences in fixation time: beginner level learners show relatively longer mean fixation durations, whereas advanced learners display comparatively shorter durations.

## Data Availability

The raw data supporting the conclusions of this article will be made available by the authors, without undue reservation.
